# Concomitant Discontinuation of Cardiovascular Therapy and Adjuvant Hormone Therapy Among Patients With Breast Cancer

**DOI:** 10.1001/jamanetworkopen.2023.23752

**Published:** 2023-07-17

**Authors:** Wei He, Erwei Zeng, Arvid Sjölander, Laila Hübbert, Elham Hedayati, Kamila Czene

**Affiliations:** 1 Chronic Disease Research Institute, The Children’s Hospital, and National Clinical Research Center for Child Health, School of Public Health, School of Medicine, Zhejiang University, Hangzhou, China; 2 Department of Nutrition and Food Hygiene, School of Public Health, Zhejiang University, Hangzhou, China; 3 Department of Medical Epidemiology and Biostatistics, Karolinska Institutet, Stockholm, Sweden; 4 Department of Cardiology, Linköping University, Norrköping, Sweden; 5 Department of Health, Medicine and Caring Sciences, Linköping University, Norrköping, Sweden; 6 Department of Oncology-Pathology, Karolinska Institutet, Stockholm, Sweden

## Abstract

**Importance:**

A large proportion of patients with breast cancer concomitantly use adjuvant hormone therapy and cardiovascular therapy.

**Objective:**

To examine the relative risk of discontinuing cardiovascular therapy during the periods before and after discontinuation of adjuvant hormone therapy.

**Design, Setting, and Participants:**

This population-based cohort study included all women aged 40 to 74 years in Stockholm, Sweden, who were diagnosed with breast cancer and concomitantly using adjuvant hormone therapy and cardiovascular therapy. Patients were enrolled from July 1, 2005, to August 31, 2020, with a median follow-up of 7.2 years. Data were analyzed from November 3, 2021, to May 12, 2022.

**Exposure:**

Discontinuation of adjuvant hormone therapy.

**Main Outcomes and Measures:**

The main outcome was discontinuation of cardiovascular therapy (cardiovascular drugs, statins, or aspirin) within 1 year before and after discontinuation of adjuvant hormone therapy. Incidence rate ratios with 95% CIs were estimated using Poisson regression. Furthermore, hazard ratios (HRs) with 95% CIs for cause-specific mortality were estimated using Cox proportional hazards regression models, comparing those who discontinued and continued adjuvant hormone therapy.

**Results:**

A total of 5493 patients with breast cancer who concomitantly used cardiovascular therapy were identified; 1811 who discontinued adjuvant hormone therapy were individually matched to 1 patient each who continued therapy by year of breast cancer diagnosis, age at diagnosis, and use of the same cardiovascular therapy. Most patients (4070 [74.1%]) were aged 60 years or older at diagnosis. At the time when patients discontinued adjuvant hormone therapy, 248 (12.2%) concomitantly discontinued their cardiovascular therapy. During follow-up, a higher discontinuation rate of cardiovascular therapy was also observed among those who discontinued adjuvant hormone therapy. Consistently, adjuvant hormone therapy discontinuation was associated with an increased risk of death not only due to breast cancer (HR, 1.43; 95 CI%, 1.01-2.01) but also cardiovascular disease (HR, 1.79; 95 CI%, 1.15-2.81). Stratifying the analyses on baseline type of adjuvant hormone therapy yielded consistent results.

**Conclusions and Relevance:**

In this cohort study of data from population-based registers in Sweden, patients who discontinued adjuvant hormone therapy were also more likely to discontinue cardiovascular therapy, especially at the time when they discontinued adjuvant hormone therapy. These findings suggest that clinicians should shift from single- to multiple-disease focus to prevent discontinuation of therapies for other diseases among patients with breast cancer.

## Key Points

**Question:**

Do patients with breast cancer tend to discontinue their cardiovascular therapy at the same time they discontinue adjuvant hormone therapy?

**Findings:**

In this population-based cohort study of 5493 patients, the highest risk of discontinuing cardiovascular therapy was observed at the time when patients discontinued adjuvant hormone therapy. Furthermore, patients who discontinued adjuvant hormone therapy were at a higher risk of both breast cancer mortality and cardiovascular mortality.

**Meaning:**

These findings suggest that multiple disease−focused interventions are warranted to prevent discontinuation of treatment for other diseases and to improve overall survival in patients with breast cancer.

## Teaser

This cohort study investigates whether patients with breast cancer who discontinue adjuvant hormone therapy tend to discontinue their cardiovascular therapy concomitantly, examines disease-specific morality by discontinuation of adjuvant hormone therapy, and identifies factors associated with discontinuation of therapy among patients with breast cancer.

## Introduction

Breast cancer is the most common female cancer, with 684 996 women dying of this disease in 2020 worldwide.^1^ Adjuvant hormone therapy reduces breast cancer mortality by 31%.^2^ Despite the known benefit, discontinuation of adjuvant hormone therapy is common, with a 5-year discontinuation rate ranging from 31% to 73%,^3,4^ which could subsequently lead to therapy failure and poor breast cancer outcomes.^5,6^

Improved survival in patients with breast cancer has brought increased incidence of comorbidities. Cardiovascular disease is one of the most frequent comorbidities among patients with breast cancer.^7,8^ Except death due to breast cancer, cardiovascular disease is the first leading cause of death in patients with breast cancer in Sweden.^9^ Pharmacological therapy, such as cardiovascular drugs, statins, and aspirin, remains the most common intervention to reduce the risk of cardiovascular diseases and mortality.^10,11,12^ Consequently, a large proportion of patients with breast cancer concurrently use multiple medications. However, nonadherence to long-term cardiovascular therapy remains a concern for the expanding population of patients who survive breast cancer.^13,14,15,16,17,18^ Despite previous studies having identified a few shared risk factors for discontinuation of adjuvant hormone therapy and cardiovascular therapy,^13,14,15^ little is known about whether discontinuation of these 2 therapies happens simultaneously.

We hypothesized that patients who discontinue adjuvant hormone therapy were more likely to discontinue cardiovascular therapy and thus had higher mortality due to cardiovascular disease. Specifically, our study aims to (1) investigate whether patients with breast cancer who discontinue adjuvant hormone therapy tend to discontinue their cardiovascular therapy concomitantly; (2) examine breast cancer–specific mortality and cardiovascular disease–specific morality by discontinuation of adjuvant hormone therapy; and (3) identify shared and unshared factors associated with discontinuation of adjuvant hormone therapy and cardiovascular therapy.

## Methods

The regional Ethical Review Authority in Stockholm, Sweden, approved this cohort study. Informed consent is not required for large-scale, registry-based studies in Sweden, provided that the study is deemed ethical by the Ethical Review Authority. This study followed the Strengthening the Reporting of Observational Studies in Epidemiology (STROBE) reporting guideline.

### Data Source and Study Population

Using unique personal identification numbers,^19^ we linked 8 Swedish health registers: the Stockholm-Gotland Quality Register for Breast Cancer,^9,20^ Prescribed Drug Register,^21^ Mammography Screening Database,^22^ National Patient Register,^23^ Longitudinal Integrated Database for Health Insurance and Labour Market Studies (LISA),^24^ Multi-Generation Register,^25^ National Cancer Register,^26^ and Cause of Death Register.^27^ The Stockholm-Gotland Quality Register for Breast Cancer (the Stockholm-Gotland Breast Cancer Register [1976-2007] and the National Quality Register for Breast Cancer [2008 onward]) includes all patients with breast cancer diagnosed in the Stockholm-Gotland region and provides detailed information on tumor characteristics and treatments, with 99.9% of incident breast cancer cases being recorded.^9,20^ The Prescribed Drug Register contains prescription records nationwide since July 2005, with 99.7% completeness.^21^ The Cause-of-Death Register (1952 onward) assesses the underlying cause of death according to strict rules by the patient’s usual physician or the physician last seeing the patient before death and records a specific underlying cause of death for 96% of individuals.^27^

Through linkage between the Stockholm-Gotland Quality Register for Breast Cancer and the Prescribed Drug Register, we identified 13 053 women diagnosed with nonmetastatic and estrogen receptor–positive breast cancer at 40 to 74 years of age from July 1, 2005, to August 31, 2020, in Stockholm, Sweden, who initiated adjuvant hormone therapy (≥2 prescriptions of tamoxifen citrate [Anatomical Therapeutic Chemical (ATC) code L02BA01] and/or aromatase inhibitors [ATC code L02BG]). Among them, we further identified 5493 patients with breast cancer who concomitantly used cardiovascular therapy, defined as filling at least 2 prescriptions of cardiovascular drugs (ATC codes C02 antihypertensives, C03 diuretics, C07 β-blocking agents, C08 calcium channel blockers, and C09 agents acting on the renin-angiotensin system), statins (ATC code C10AA), or aspirin (ATC code B01AC06) after adjuvant hormone therapy initiation and before end of follow-up (eFigure in Supplement 1).

### Discontinuation of Adjuvant Hormone Therapy and Cardiovascular Therapy

Discontinuation of adjuvant hormone therapy and cardiovascular therapy was defined as failure to refill a prescription of the corresponding therapy within 6 months after a previous prescription.^3^ Swedish pharmacies dispense a maximum of 3 months of prescribed drugs. A 6-month gap indicated at least 2 missed drug dispensations, suggesting treatment discontinuation. Patients were followed up from the first prescription of the corresponding therapy until treatment discontinuation, local recurrence, distant metastasis, second primary breast cancer (>3 months after a primary breast cancer diagnosis), death, emigration, completion of 5-year adjuvant hormone therapy, or end of the study period (August 31, 2020), whichever came first. Analyses for cardiovascular therapy discontinuation were additionally censored at occurrence or recurrence of cardiovascular comorbidities after start of follow-up, including heart failure (*International Statistical Classification of Diseases and Related Health Problems, Tenth Revision* [*ICD-10*] code I50), arrhythmias (*ICD-10* codes I47-I49), ischemic heart disease (*ICD-10* codes I20-I25), and cerebrovascular diseases (*ICD-10* codes I60-I69).

### Breast Cancer Mortality and Cardiovascular Disease–Specific Mortality

The main underlying cause of death was used to identify death due to breast cancer through *ICD-10* code C50 and death due to cardiovascular disease through *ICD-10* codes I00 to I99 in the Cause-of-Death Register. Patients were followed up until death, emigration, or end of the study period (December 31, 2019; last date cause of death was available), whichever came first. The median follow-up time was 6.5 years.

### Factors Associated With Discontinuation of Adjuvant Hormone Therapy and Cardiovascular Therapy

Information on divorce and income at diagnosis was retrieved from the LISA register.^24^ Use of symptom-relieving drugs, including analgesics (ATC code N02), gastrointestinal tract drugs (ATC codes A02, A03, A04, A06, and A07), antidepressants (ATC code N06A), or hypnotics and/or sedatives (ATC code N05C), was defined as having 1 prescription of corresponding drugs during the first 6 months of adjuvant hormone therapy. Prediagnosis hormone replacement therapy was defined as a systematic use of estrogen and/or progesterone (ATC codes G03C, G03D, and G03F) with at least 1 prescription within 6 months before breast cancer diagnosis, excluding patch and vaginal cream preparations. Participation in mammography screening was defined as attendance of mammography within 2 years before breast cancer diagnosis. Family history of breast cancer and major cardiovascular events was obtained by linking the Multi-Generation Register to the National Cancer Register and National Patient Register, respectively. Charlson Comorbidity Index score before breast cancer diagnosis was calculated using main diagnoses retrieved from the Swedish Patient Register.^28^

### Statistical Analysis

Data were analyzed from November 3, 2021, to May 12, 2022. To investigate whether adjuvant hormone therapy and cardiovascular therapy tend to be discontinued concomitantly, we first identified 1811 patients who discontinued adjuvant hormone therapy and used cardiovascular therapy within 1 year before the therapy discontinuation. We compared the discontinuation rate of cardiovascular therapy at adjuvant hormone therapy discontinuation with other periods (ie, 1 year before and after the therapy discontinuation). Poisson regression with a clustered sandwich estimator was used to account for lack of independence due to patients using more than 1 cardiovascular therapy.^29^

To further investigate the association between discontinuation of adjuvant hormone therapy and cardiovascular therapy, we used a matched cohort. For each patient who discontinued adjuvant hormone therapy, we randomly sampled 1 patient who continued the therapy at the first patient’s discontinuation date and used the same cardiovascular medication. Those who discontinued and continued medications were individually matched on year of breast cancer diagnosis and age at diagnosis (±3 years). We used χ^2^ tests to compare baseline characteristics between those who continued and discontinued adjuvant hormone therapy. Poisson regression with clustered sandwich estimator was used to estimate the association, adjusting for matching variables, tumor size, lymph node status, tumor grade, progesterone receptor status, surgery type, chemotherapy, radiotherapy, baseline hormone therapy type, prediagnosis use of cardiovascular therapy, prediagnosis major cardiovascular event, and Charlson Comorbidity Index score at diagnosis. This analysis was stratified by 4 time bands in relation to adjuvant hormone therapy discontinuation: −12 to −3 months, −3 months, 3 months, and 3 to 12 months.

Kaplan-Meier curves were further constructed for breast cancer–specific and cardiovascular disease–specific mortality for those who discontinued adjuvant hormone therapy and matched patients who continued therapy. Marginal Cox proportional hazards regression models with clustered sandwich estimator were used to compare cause-specific mortality between patients who discontinued and matched patients who continued therapy, adjusting for matching variables, tumor size, lymph node status, tumor grade, progesterone receptor status, surgery type, chemotherapy, radiotherapy, baseline hormone therapy type, prediagnosis use of cardiovascular therapy, prediagnosis major cardiovascular event, and Charlson Comorbidity Index score at diagnosis. All the aforementioned analyses were stratified by baseline type of adjuvant hormone therapy.

Finally, we identified shared and unshared factors associated with discontinuation of adjuvant hormone therapy and cardiovascular therapy among patients who concomitantly used these 2 therapies. Marginal Cox proportional hazards regression models with clustered sandwich estimator were used, adjusting for age at breast cancer diagnosis, diagnosis calendar period, tumor size, lymph node status, tumor grade, progesterone receptor status, surgery type, chemotherapy, radiotherapy, baseline hormone therapy type, prediagnosis use of cardiovascular therapy, prediagnosis major cardiovascular event, and Charlson Comorbidity Index score at diagnosis. All analyses were performed using SAS, version 9.4 (SAS Institute Inc); Stata, version 17.0 (StataCorp LLC); or R, version 1.4.1106 (R Project for Statistical Computing) at a 2-tailed α = .05.

## Results

### Concomitant Use of Cardiovascular Therapy and Adjuvant Hormone Therapy

Of the 13 053 patients who initiated adjuvant hormone therapy, 5493 (42.1%) concomitantly used at least 1 of the following cardiovascular therapies: 4966 (38.0%) used cardiovascular drugs, 1861 (14.3%) used statins, and 968 (7.4%) used aspirin. In the full cohort of patients who concomitantly used cardiovascular therapies, 4070 patients (74.1%) were diagnosed after 60 years of age and 4046 of 5176 (78.2%) used the corresponding cardiovascular therapy before breast cancer diagnosis (Table 1). Table 1 shows baseline characteristics, comorbidities, clinical features, and treatment information by discontinuation of adjuvant hormone therapy in the matched cohort. Most variables presented in Table 1 had a low level of missingness (<5%) and the missingness was not associated with adjuvant hormone therapy discontinuation, except for tumor grade, with data missing for 395 patients (7.2%). Among women who concomitantly used both therapies, the 5-year cumulative discontinuation rate was 42.1% for adjuvant hormone therapy and 47.6% for cardiovascular therapy.

**Table 1.** Baseline Characteristics of Women Diagnosed With Breast Cancer Who Concomitantly Used Cardiovascular Therapy in Stockholm, Sweden, 2005 to 2020^a^

Characteristic	Full cohort (N = 5493)	Matched cohort by continuation of adjuvant hormone therapy^b^		
		Discontinued (n = 2704)	Continued (n = 2696)	*P* value
Age at diagnosis, y				
40-49	327 (6.0)	114 (4.3)	96 (3.6)	.46
50-59	1096 (20.0)	436 (16.1)	438 (16.2)	
60-74	4070 (74.1)	2154 (79.7)	2162 (80.2)	
Tumor size, mm				
≤20	3651 (66.5)	1829 (67.6)	1816 (67.4)	.87
>20	1835 (33.4)	872 (32.2)	874 (32.4)	
Unknown	7 (0.1)	3 (0.1)	6 (0.2)	
Lymph node involvement				
Negative	3780 (68.8)	1857 (68.7)	1897 (70.4)	.29
Positive	1555 (28.3)	780 (28.8)	747 (27.7)	
Unknown	158 (2.9)	67 (2.5)	52 (19.3)	
Elston–Ellis tumor grade^c^				
1	1089 (19.8)	606 (22.4)	549 (20.4)	<.001
2	2917 (53.1)	1336 (49.4)	1535 (56.9)	
3	1092 (19.9)	550 (20.3)	477 (17.7)	
Unknown	395 (7.2)	212 (7.8)	135 (5.0)	
Progesterone receptor status				
Positive	4480 (81.6)	2219 (82.1)	2211 (82.0)	.72
Negative	915 (16.6)	434 (16.0)	444 (16.5)	
Unknown	98 (1.8)	51 (1.9)	41 (1.5)	
Surgery type				
Lumpectomy	3961 (72.1)	1896 (70.1)	1985 (73.6)	.007
Mastectomy	1399 (25.5)	741 (27.4)	665 (24.7)	
No surgery	133 (2.4)	67 (2.5)	46 (1.7)	
Chemotherapy				
No	3391 (61.7)	1783 (65.9)	1809 (67.1)	.45
Yes	2031 (37.0)	881 (32.6)	855 (31.7)	
Unknown	71 (1.3)	40 (1.5)	32 (1.2)	
Radiotherapy				
No	754 (13.7)	415 (15.4)	384 (14.2)	.23
Yes	4664 (85.0)	2251 (83.2)	2282 (84.6)	
Unknown	75 (1.4)	38 (1.4)	30 (1.1)	
Baseline therapy type				
Aromatase inhibitors	3282 (59.7)	1572 (58.1)	1520 (56.4)	.19
Tamoxifen	2211 (40.3)	1132 (41.9)	1176 (43.6)	
Mammography screening				
Nonparticipation	671 (12.2)	350 (12.9)	248 (9.2)	<.001
Participation	4064 (74.0)	1859 (68.8)	2007 (74.2)	
Unknown	758 (13.8)	495 (18.3)	441 (16.3)	
Divorce				
No	4183 (76.2)	2004 (74.1)	2068 (76.7)	.03
Yes	1305 (23.8)	699 (25.9)	627 (23.3)	
Unknown	5 (<0.1)	1 (<0.1)	1 (<0.1)	
Income^d^				
Low	2744 (50.0)	1593 (58.9)	1475 (54.7)	.002
High	2744 (50.0)	1110 (41.1)	1220 (45.3)	
Unknown	5 (<0.1)	1 (<0.1)	1 (<0.1)	
Family history of breast cancer^e^				
No	4198 (84.5)	2060 (87.2)	2103 (85.3)	.06
Yes	771 (15.5)	303 (12.8)	363 (14.7)	
Family history of major cardiovascular event^f^				
No	2145 (41.1)	1035 (41.1)	1001 (38.5)	.06
Yes	3073 (58.9)	1481 (58.9)	1596 (61.5)	
Hormone replacement therapy^g^				
No	4165 (90.1)	2019 (86.5)	2137 (90.6)	<.001
Yes	459 (9.9)	314 (13.5)	223 (9.4)	
Symptom-relieving drugs				
Analgesics				
No	3845 (70.0)	1716 (63.5)	1934 (71.7)	<.001
Yes	1648 (30.0)	988 (36.5)	762 (28.3)	
Gastrointestinal tract drugs				
No	3719 (67.7)	1691 (62.5)	1937 (71.8)	<.001
Yes	1774 (32.3)	1013 (37.5)	759 (28.2)	
Antidepressants				
No	4563 (83.1)	2153 (79.6)	2311 (85.7)	<.001
Yes	930 (16.9)	551 (20.4)	385 (14.3)	
Hypnotics and sedatives				
No	4060 (73.9)	1833 (67.8)	2063 (76.5)	<.001
Yes	1433 (26.1)	871 (32.2)	633 (23.5)	
Prediagnosis use of cardiovascular therapy^h^				
No	1130 (21.8)	355 (14.2)	377 (15.1)	.36
Yes	4046 (78.2)	2151 (85.8)	2123 (84.9)	
Charlson Comorbidity Index score				
0	3986 (72.6)	1784 (66.0)	1924 (71.4)	<.001
1	810 (14.7)	512 (18.9)	415 (15.4)	
≥2	697 (12.7)	408 (15.1)	357 (13.2)	
Prediagnosis major cardiovascular event^i^				
No	4917 (89.5)	2201 (81.4)	2317 (85.9)	<.001
Yes	576 (10.5)	503 (18.6)	379 (14.1)	
Prediagnosis deep vein thrombosis				
No	5326 (97.0)	2621 (96.9)	2638 (97.8)	.03
Yes	167 (3.0)	83 (3.1)	58 (2.2)	
Prediagnosis osteoporosis				
No	5388 (98.1)	2647 (97.9)	2654 (98.4)	.13
Yes	105 (1.9)	57 (2.1)	42 (1.6)	

^a^ Unless otherwise indicated, data are expressed as No. (%) of patients. Percentages may not total 100 owing to rounding.

^b^ Patients who discontinued adjuvant hormone therapy were matched 1:1 with those who continued therapy on year of breast cancer diagnosis, age at diagnosis (±3 years), and type of cardiovascular therapy.

^c^ Grade 1 indicates a well-differentiated state wherein the cancer cells grow slowly and resemble normal breast cells. Grade 2 indicates a moderately differentiated state wherein the cancer cells grow at an intermediate rate and exhibit characteristics between grades 1 and 3. Grade 3 indicates a poorly differentiated state wherein the cancer cells significantly differ from normal breast cells and tend to grow and spread rapidly.

^d^ Categorized as low and high using the median as a cutoff.

^e^ Defined as having a female first-degree relative with breast cancer before the index patient’s breast cancer diagnosis. Analysis was restricted to patients who were registered in the Swedish Multi-Generation Register.

^f^ Defined as having a first-degree relative with ischemic heart diseases (*International Statistical Classification of Diseases and Related Health Problems, Tenth Revision* [*ICD-10*] codes I20-I25) or cerebrovascular diseases (*ICD-10* codes I60-I69) before the index patient’s breast cancer diagnosis. Analysis was restricted to patients who were registered in the Swedish Multi-Generation Register.

^g^ Analysis was restricted to postmenopausal women.

^h^ Defined as at least 1 prescription of cardiovascular drugs (Anatomical Therapeutic Chemical [ATC] codes C02, C03, C07, C08, and C09), statins (ATC code C10AA), or aspirin (ATC code B01AC06) within 1 year before breast cancer diagnosis. Analysis was restricted to patients diagnosed after July 1, 2006.

^i^ Defined as having a diagnosis of ischemic heart diseases (*ICD-10* codes I20-I25) or cerebrovascular diseases (*ICD-10* codes I60-I69) before breast cancer diagnosis.

### Concomitant Discontinuation of Cardiovascular Therapy and Adjuvant Hormone Therapy

At discontinuation of adjuvant hormone therapy, 248 of 2030 patients (12.2%) also discontinued their cardiovascular therapy (135 of 1336 [10.1%] for cardiovascular drugs, 71 of 438 [16.2%] for statins, and 42 of 256 [16.4%] for aspirin). The discontinuation rate of cardiovascular therapy was the highest at the time of discontinuing adjuvant hormone therapy compared with other periods of follow-up, with an indcidence rate ratio (IRR) of 197.63 (95% CI, 169.11-230.97). Stratified analyses by baseline type of adjuvant hormone therapy yielded consistent results, with IRRs of 199.35 (95% CI, 162.47-244.60) and 194.81 (95% CI, 153.28-247.60) for aromatase inhibitors users and tamoxifen users, respectively.

During the period 1 year before and after discontinuation of adjuvant hormone therapy, the incidence of discontinuing cardiovascular therapy was 0.82 per 1000 person-days among discontinuers and 0.41 per 1000 person-days among continuers. Discontinuers were more likely to discontinue cardiovascular therapy, with IRRs of 1.83 (95% CI, 1.41-2.37) and 2.31 (95% CI, 1.74-3.05) within 3 months before and after discontinuation, respectively (Table 2). Similar associations were observed for aromatase inhibitor users and tamoxifen users (eTable 1 in Supplement 1).

**Table 2.** IRR of Discontinuing Cardiovascular Therapy Before and After Discontinuation of Adjuvant Hormone Therapy in Patients With Breast Cancer^a^

Patient group	Time in relation to adjuvant hormone therapy discontinuation			
	−365 to −91 d	−90 to −1 d	1 to 90 d	91 to 365 d
Discontinued adjuvant hormone therapy				
No. of events	292	173	144	153
Follow-up time, d	547 482	192 166	151 335	341 726
Incidence rate (per 1000 person-days)	0.53	0.90	0.95	0.45
Continued adjuvant hormone therapy				
No. of events	240	104	86	178
Follow-up time, d	579 181	212 848	206 736	505 065
Incidence rate (per 1000 person-days)	0.41	0.49	0.42	0.35
IRR (95% CI)^b^	1.29 (1.08-1.54)	1.83 (1.41-2.37)	2.31 (1.74-3.05)	1.28 (1.01-1.62)

Abbreviation: IRR, incidence rate ratio.

^a^ Patients who discontinued adjuvant hormone therapy were matched 1:1 with those who continued therapy by year of breast cancer diagnosis, age at diagnosis (±3 years), and type of cardiovascular therapy. Patients were followed up from 1 year before adjuvant hormone therapy discontinuation until discontinuation of cardiovascular therapy, 1 year after adjuvant hormone therapy discontinuation, local recurrence, distant metastasis, contralateral breast cancer, cardiovascular comorbidities, death, emigration, completion of 5-year adjuvant hormone therapy, or end of the study period (August 31, 2020), whichever came first.

^b^ Adjusted for matching variables, tumor size, lymph node status, tumor grade, progesterone receptor status, surgery type, chemotherapy, radiotherapy, baseline hormone therapy type, prediagnosis use of cardiovascular therapy, prediagnosis major cardiovascular event, prediagnosis menopausal symptoms, and Charlson Comorbidity Index at diagnosis.

### Cause-Specific Mortality by Discontinuation of Adjuvant Hormone Therapy Among Patients Who Concomitantly Used Cardiovascular Therapy

**Figure 1.**
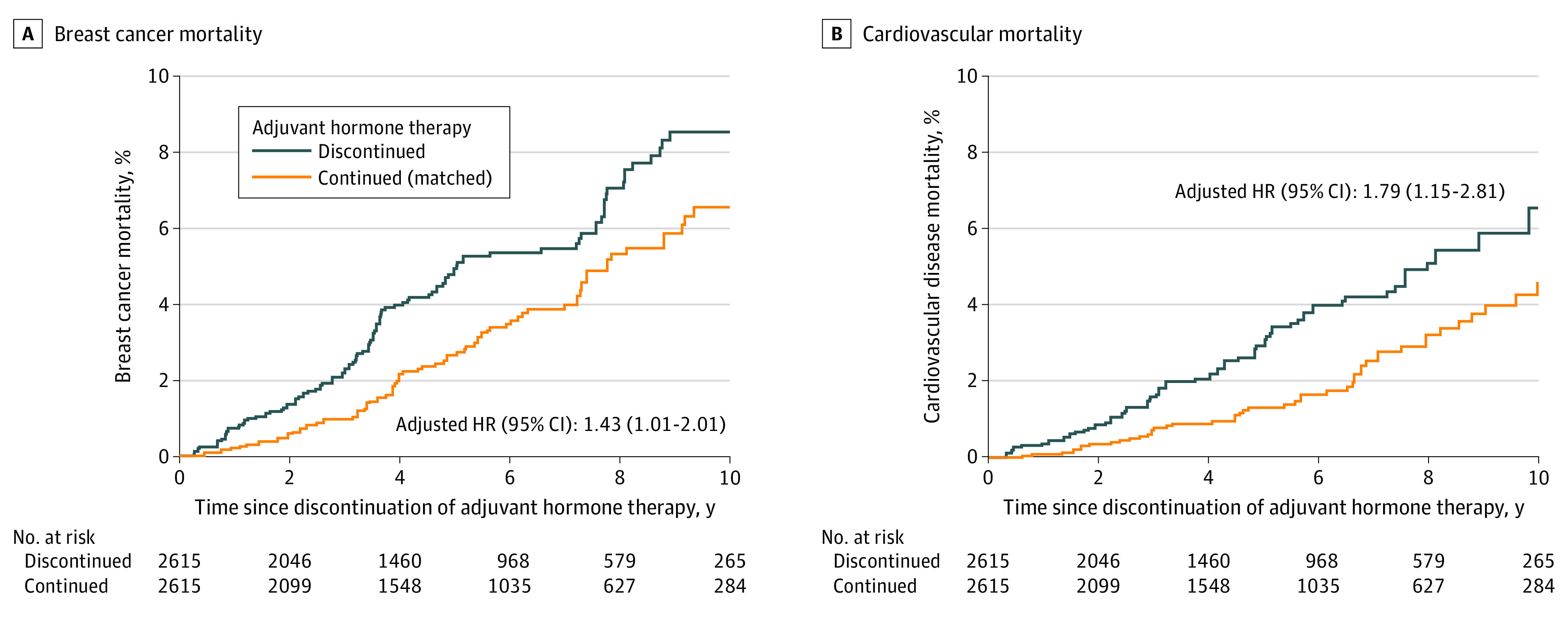
Cause-Specific Mortality by Discontinuation of Adjuvant Hormone Therapy Among Patients With Breast Cancer Patients who discontinued adjuvant hormone therapy were 1:1 matched to those who continued therapy by year of breast cancer diagnosis, age at diagnosis (±3 years), and type of cardiovascular therapy. Patients were followed up until death, emigration, or end of the study period (December 31, 2019), whichever came first. Hazard ratios (HRs) were adjusted for matching variables, tumor size, lymph node status, tumor grade, progesterone receptor status, surgery type, chemotherapy, radiotherapy, baseline hormone therapy type, prediagnosis use of cardiovascular therapy, prediagnosis major cardiovascular event, and Charlson Comorbidity Index score at diagnosis.

Figure 1 presents the cause-specific mortality by discontinuation of adjuvant hormone therapy. In addition to a higher risk of death due to breast cancer (HR, 1.43 [95 CI%, 1.01-2.01]), a higher risk of death due to cardiovascular disease (HR, 1.79 [95 CI%, 1.15-2.81]) was observed among patients who discontinued adjuvant hormone therapy, compared with matched patients who continued therapy. Similar associations were observed for patients who used aromatase inhibitors and tamoxifen (eTable 2 in Supplement 1).

### Factors Associated With Discontinuation of Cardiovascular Therapy and Discontinuation of Adjuvant Hormone Therapy

**Figure 2.**
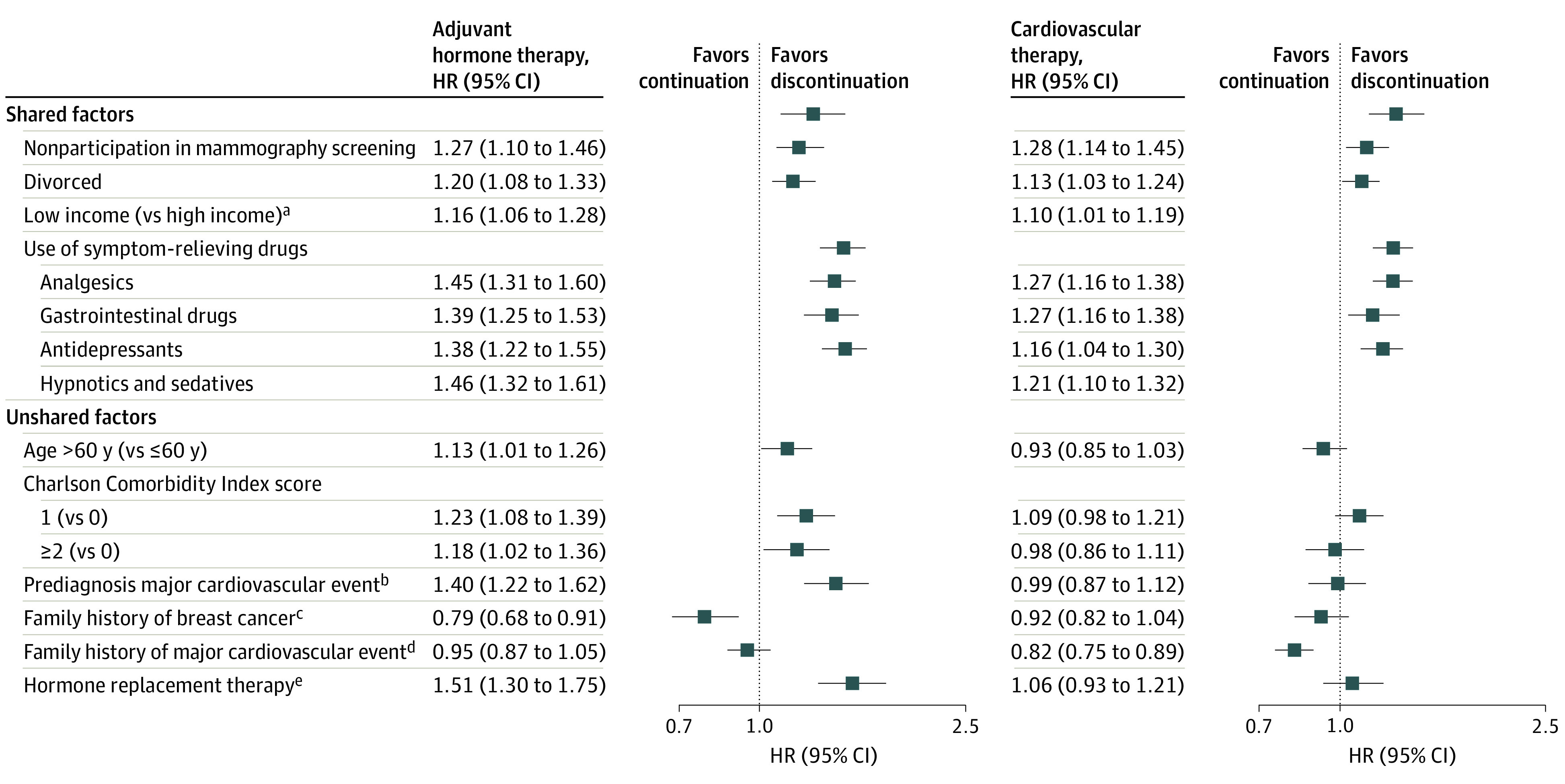
Factors Associated With Discontinuation of Cardiovascular Therapy and Adjuvant Hormone Therapy Among Patients With Breast Cancer Shared factors were statistically significant for discontinuation of both therapies with same direction of association; unshared factors were statistically significant only for discontinuation of one of the therapies. Hazard ratios (HRs) were adjusted for age at diagnosis, calendar period of diagnosis, tumor size, lymph node status, tumor grade, progesterone receptor status, surgery type, chemotherapy, radiotherapy, baseline hormone therapy type, prediagnosis use of cardiovascular therapy, prediagnosis major cardiovascular event, and Charlson Comorbidity Index score at diagnosis. ^a^Categorized as low and high using the median as a cutoff. ^b^Defined as having a diagnosis of ischemic heart diseases (*International Statistical Classification of Diseases and Related Health Problems, Tenth Revision* [*ICD-10*] codes I20-I25) or cerebrovascular diseases (*ICD-10* codes I60-I69) before breast cancer diagnosis. ^c^Defined as having a female first-degree relative with breast cancer before index patient’s breast cancer diagnosis. ^d^Defined as having a first-degree relative with ischemic heart diseases (*ICD-10* codes I20-I25) or cerebrovascular diseases (*ICD-10* codes I60-I69) before index patient’s breast cancer diagnosis. ^e^Analysis was restricted to postmenopausal women.

Figure 2 shows factors associated with discontinuation of adjuvant hormone therapy and cardiovascular therapy. Shared risk factors included nonparticipation in mammography screening, low income, divorce, and use of symptom-relieving drugs including analgesics, gastrointestinal tract drugs, antidepressants, and hypnotics and/or sedatives. Unshared risk factors included age at diagnosis, family history of breast cancer, family history of major cardiovascular events, use of hormone replacement therapy, baseline Charlson Comorbidity Index score, and prediagnosis major cardiovascular events.

## Discussion

Our findings suggest that patients with breast cancer who discontinued adjuvant hormone therapy were also more likely to discontinue their cardiovascular therapy. In particular, patients with breast cancer tended to discontinue these 2 therapies at the same time. Moreover, those who discontinued adjuvant hormone therapy were at a higher mortality risk due not only to breast cancer but also cardiovascular disease.

Our findings further suggest that more than 2 in 5 patients with breast cancer concurrently used cardiovascular therapy and adjuvant hormone therapy. Many patients with breast cancer, especially elderly patients, present with multiple chronic diseases and concomitantly use several long-term medications.^30^ Elderly patients now account for most patients who survive breast cancer, and the number is projected to double by 2040.^31,32^ Such a large number highlights the clinical importance of monitoring use of cardiovascular medications among patients with breast cancer.

We also found that among concomitant users of adjuvant hormone therapy and cardiovascular therapy, the discontinuation of these 2 therapies tends to happen concomitantly. More than 1 in 9 patients with breast cancer discontinued their cardiovascular therapy when they discontinued their adjuvant hormone therapy. This finding suggests that, once oncologists detected discontinuation of adjuvant hormone therapy, they should ask patients about their adherence to cardiovascular therapy. That is, oncologists should shift from single- to multiple-disease focus to prevent discontinuation of treatment by patients with breast cancer, which is likely to be achieved through an extended cardio-oncology approach and cooperation.

In addition to concomitant discontinuation, we found that patients who discontinued adjuvant hormone therapy were also more likely to discontinue cardiovascular therapy in other time periods, both before and after adjuvant hormone therapy discontinuation. This result is consistent with results of a previous study^33,34^ showing that nonadherence to cardiovascular therapy before breast cancer diagnosis is associated with nonadherence to adjuvant hormone therapy.

As expected, we found that adjuvant hormone therapy discontinuation was associated with an increased mortality not only due to breast cancer but also cardiovascular disease, after adjusting for tumor characteristics, treatments, prediagnosis major cardiovascular event, and prediagnosis use of cardiovascular therapy. Our result suggests that the concomitant discontinuation is highly likely to translate to higher mortality. This aligns with previous studies^35,36^ indicating that nonadherence to cardiovascular medications following breast cancer diagnosis is associated with a higher level of low-density lipoprotein and an increased risk of a cardiac event. To potentially prevent these unnecessary deaths, interventions focusing on multiple medications adherence are urgently needed. Furthermore, while the association with cause-specific mortality did not significantly differ by hormone therapy type, it is important for future studies with a larger sample size to replicate this analysis considering the difference in efficacy and adverse effects profile between tamoxifen and aromatase inhibitors.^37,38^

Discontinuation of adjuvant hormone therapy and cardiovascular therapy may share common risk factors and underlying mechanisms. In our study, we found that nonparticipation in mammography screening, divorce, low income, and use of symptom-relieving drugs are associated with both discontinuation of adjuvant hormone therapy and discontinuation of cardiovascular therapy. These shared factors may serve as potential targets for interventions that may simultaneously improve adherence to more than 1 life-saving medication, thus having greater cost-effectiveness. For example, we found that patients who used symptom-relieving drugs were at higher risk of discontinuing both therapies, suggesting that only prescribing symptom-relieving drugs seems to be not enough to prevent therapy discontinuation once patients have therapy-related symptoms. Other additional interventions, such as better clinician-patient communication, are needed to improve adherence for both adjuvant hormone therapy and cardiovascular therapy.

Despite that, we also identified several unique factors specifically associated with discontinuation of adjuvant hormone therapy or discontinuation of cardiovascular therapy. For example, women with a family history of breast cancer were less likely to discontinue their adjuvant hormone therapy, but no difference was found for discontinuation of cardiovascular therapy. These unique factors suggest that discontinuation of different therapies also has its own unique mechanism and thus should be investigated separately to build a full picture of discontinuation of specific therapy.

### Limitations

This study has several limitations. First, using prescription data from the register may underestimate the discontinuation rate, because prescription refill does not necessarily guarantee that a patient used the drug. Second, misclassifying patients who used aspirin as nonusers is possible because the Swedish Prescribed Drug Register does not capture over-the-counter medications. However, given the fact that low-dose and/or long-term use of aspirin is prescribed, we anticipate this misclassification is likely minimal. Third, although we censored patients at disease progress of both breast cancer and cardiovascular disease and adjusted for Charlson Comorbidity Index score at diagnosis and cancer treatments, we cannot rule out the possibility that the concomitant discontinuation of adjuvant hormone therapy and cardiovascular therapy could have been a result of clinical decision-making. While this may partially contribute to the observed association in our study, it is unlikely to substantially account for all associations we have reported. Fourth, the study did not have sufficient power to conduct a stratification analysis by timing of initiating cardiovascular therapy due to limited sample size for patients who initiated cardiovascular therapy after breast cancer diagnosis. Fifth, caution is needed when generalizing our findings to other countries, since our study population included only patients with breast cancer diagnosed in Sweden.

## Conclusions

In this cohort study of patients with breast cancer concomitantly using adjuvant hormone therapy and cardiovascular therapy, our results call for extra vigilance for concomitant discontinuation of adjuvant hormone therapy and cardiovascular therapy in clinical practice. Shared risk factors identified in our study may help to develop targeted interventions to simultaneously improve adherence to both therapies and subsequently improve overall survival among patients with breast cancer.

## Notes

Supplement 1. **eFigure.** Flowchart of Study Population **eTable 1.** Incidence Rate Ratio and 95% CI of Discontinuing Cardiovascular Therapy Before and After Discontinuation of Adjuvant Hormone Therapy in Breast Cancer Patients, Stratified by Baseline Type of Adjuvant Hormone Therapy **eTable 2.** Cause-Specific Mortality by Discontinuation of Adjuvant Hormone Therapy in Breast Cancer Patients, Stratified by Baseline Type of Adjuvant Hormone Therapy

Supplement 2.
Data Sharing Statement

## References

[1] Sung H, Ferlay J, Siegel RL, . Global Cancer Statistics 2020: GLOBOCAN estimates of incidence and mortality worldwide for 36 cancers in 185 countries. CA Cancer J Clin. 2021;71(3):209-249. doi:10.3322/caac.21660 33538338

[2] Early Breast Cancer Trialists’ Collaborative Group (EBCTCG). Effects of chemotherapy and hormonal therapy for early breast cancer on recurrence and 15-year survival: an overview of the randomised trials. Lancet. 2005;365(9472):1687-1717. doi:10.1016/S0140-6736(05)66544-0 15894097

[3] He W, Fang F, Varnum C, Eriksson M, Hall P, Czene K. Predictors of discontinuation of adjuvant hormone therapy in patients with breast cancer. J Clin Oncol. 2015;33(20):2262-2269. doi:10.1200/JCO.2014.59.3673 26033800

[4] Murphy CC, Bartholomew LK, Carpentier MY, Bluethmann SM, Vernon SW. Adherence to adjuvant hormonal therapy among breast cancer survivors in clinical practice: a systematic review. Breast Cancer Res Treat. 2012;134(2):459-478. doi:10.1007/s10549-012-2114-5 22689091 PMC3607286

[5] Hershman DL, Shao T, Kushi LH, . Early discontinuation and non-adherence to adjuvant hormonal therapy are associated with increased mortality in women with breast cancer. Breast Cancer Res Treat. 2011;126(2):529-537. doi:10.1007/s10549-010-1132-4 20803066 PMC3462663

[6] Pistilli B, Paci A, Ferreira AR, . Serum detection of nonadherence to adjuvant tamoxifen and breast cancer recurrence risk. J Clin Oncol. 2020;38(24):2762-2772. doi:10.1200/JCO.19.01758 32568632 PMC7430219

[7] Edwards BK, Noone AM, Mariotto AB, . Annual report to the nation on the status of cancer, 1975-2010, featuring prevalence of comorbidity and impact on survival among persons with lung, colorectal, breast, or prostate cancer. Cancer. 2014;120(9):1290-1314. doi:10.1002/cncr.28509 24343171 PMC3999205

[8] Strongman H, Gadd S, Matthews A, . Medium and long-term risks of specific cardiovascular diseases in survivors of 20 adult cancers: a population-based cohort study using multiple linked UK electronic health records databases. Lancet. 2019;394(10203):1041-1054. doi:10.1016/S0140-6736(19)31674-5 31443926 PMC6857444

[9] Colzani E, Liljegren A, Johansson AL, . Prognosis of patients with breast cancer: causes of death and effects of time since diagnosis, age, and tumor characteristics. J Clin Oncol. 2011;29(30):4014-4021. doi:10.1200/JCO.2010.32.6462 21911717

[10] Baigent C, Blackwell L, Collins R, ; Antithrombotic Trialists’ (ATT) Collaboration. Aspirin in the primary and secondary prevention of vascular disease: collaborative meta-analysis of individual participant data from randomised trials. Lancet. 2009;373(9678):1849-1860. doi:10.1016/S0140-6736(09)60503-1 19482214 PMC2715005

[11] Baigent C, Blackwell L, Emberson J, ; Cholesterol Treatment Trialists’ (CTT) Collaboration. Efficacy and safety of more intensive lowering of LDL cholesterol: a meta-analysis of data from 170,000 participants in 26 randomised trials. Lancet. 2010;376(9753):1670-1681. doi:10.1016/S0140-6736(10)61350-5 21067804 PMC2988224

[12] Law MR, Morris JK, Wald NJ. Use of blood pressure lowering drugs in the prevention of cardiovascular disease: meta-analysis of 147 randomised trials in the context of expectations from prospective epidemiological studies. BMJ. 2009;338:b1665. doi:10.1136/bmj.b1665 19454737 PMC2684577

[13] Calip GS, Elmore JG, Boudreau DM. Characteristics associated with nonadherence to medications for hypertension, diabetes, and dyslipidemia among breast cancer survivors. Breast Cancer Res Treat. 2017;161(1):161-172. doi:10.1007/s10549-016-4043-1 27826756 PMC7909611

[14] Calip GS, Xing S, Jun DH, Lee WJ, Hoskins KF, Ko NY. Polypharmacy and adherence to adjuvant endocrine therapy for breast cancer. J Oncol Pract. 2017;13(5):e451-e462. doi:10.1200/JOP.2016.018317 28287854

[15] Yang J, Neugut AI, Wright JD, Accordino M, Hershman DL. Nonadherence to oral medications for chronic conditions in breast cancer survivors. J Oncol Pract. 2016;12(8):e800-e809. doi:10.1200/JOP.2016.011742 27407167

[16] Brookhart MA, Patrick AR, Schneeweiss S, . Physician follow-up and provider continuity are associated with long-term medication adherence: a study of the dynamics of statin use. Arch Intern Med. 2007;167(8):847-852. doi:10.1001/archinte.167.8.847 17452550

[17] Krousel-Wood M, Thomas S, Muntner P, Morisky D. Medication adherence: a key factor in achieving blood pressure control and good clinical outcomes in hypertensive patients. Curr Opin Cardiol. 2004;19(4):357-362. doi:10.1097/01.hco.0000126978.03828.9e 15218396

[18] Packard KA, Hilleman DE. Adherence to therapies for secondary prevention of cardiovascular disease: a focus on aspirin. Cardiovasc Ther. 2016;34(6):415-422. doi:10.1111/1755-5922.12211 27473898

[19] Ludvigsson JF, Otterblad-Olausson P, Pettersson BU, Ekbom A. The Swedish personal identity number: possibilities and pitfalls in healthcare and medical research. Eur J Epidemiol. 2009;24(11):659-667. doi:10.1007/s10654-009-9350-y 19504049 PMC2773709

[20] Löfgren L, Eloranta S, Krawiec K, Asterkvist A, Lönnqvist C, Sandelin K; Steering Group of the National Register for Breast Cancer. Validation of data quality in the Swedish National Register for Breast Cancer. BMC Public Health. 2019;19(1):495. doi:10.1186/s12889-019-6846-6 31046737 PMC6498669

[21] Wettermark B, Hammar N, Fored CM, . The new Swedish Prescribed Drug Register—opportunities for pharmacoepidemiological research and experience from the first six months. Pharmacoepidemiol Drug Saf. 2007;16(7):726-735. doi:10.1002/pds.1294 16897791

[22] Lind H, Svane G, Kemetli L, Törnberg S. Breast cancer screening program in Stockholm County, Sweden—aspects of organization and quality assurance. Breast Care (Basel). 2010;5(5):353-357. doi:10.1159/000321255 21779220 PMC3132962

[23] Ludvigsson JF, Andersson E, Ekbom A, . External review and validation of the Swedish national inpatient register. BMC Public Health. 2011;11(1):450. doi:10.1186/1471-2458-11-450 21658213 PMC3142234

[24] Ludvigsson JF, Svedberg P, Olén O, Bruze G, Neovius M. The longitudinal integrated database for health insurance and labour market studies (LISA) and its use in medical research. Eur J Epidemiol. 2019;34(4):423-437. doi:10.1007/s10654-019-00511-8 30929112 PMC6451717

[25] Ekbom A. The Swedish Multi-generation Register. In: Dillner J, ed. Methods in Biobanking. Humana Press; 2011:215-220. doi:10.1007/978-1-59745-423-0_10 20949391

[26] Barlow L, Westergren K, Holmberg L, Talbäck M. The completeness of the Swedish Cancer Register: a sample survey for year 1998. Acta Oncol. 2009;48(1):27-33. doi:10.1080/02841860802247664 18767000

[27] Brooke HL, Talbäck M, Hörnblad J, . The Swedish cause of death register. Eur J Epidemiol. 2017;32(9):765-773. doi:10.1007/s10654-017-0316-1 28983736 PMC5662659

[28] Charlson ME, Pompei P, Ales KL, MacKenzie CR. A new method of classifying prognostic comorbidity in longitudinal studies: development and validation. J Chronic Dis. 1987;40(5):373-383. doi:10.1016/0021-9681(87)90171-8 3558716

[29] Ying GS, Liu C. Statistical analysis of clustered data using SAS system. Northeast SAS Users Group 2006. January 2006. Accessed January 10, 2022. https://www.lexjansen.com/nesug/nesug06/an/da01.pdf

[30] Topaloğlu US, Özaslan E. Comorbidity and polypharmacy in patients with breast cancer. Breast Cancer. 2020;27(3):477-482. doi:10.1007/s12282-019-01040-8 31898155

[31] Bluethmann SM, Mariotto AB, Rowland JH. Anticipating the “silver tsunami”: prevalence trajectories and comorbidity burden among older cancer survivors in the United States. Cancer Epidemiol Biomarkers Prev. 2016;25(7):1029-1036. doi:10.1158/1055-9965.EPI-16-0133 27371756 PMC4933329

[32] Shapiro CL. Cancer survivorship. N Engl J Med. 2018;379(25):2438-2450. doi:10.1056/NEJMra1712502 30575480

[33] Neugut AI, Zhong X, Wright JD, Accordino M, Yang J, Hershman DL. Nonadherence to medications for chronic conditions and nonadherence to adjuvant hormonal therapy in women with breast cancer. JAMA Oncol. 2016;2(10):1326-1332. doi:10.1001/jamaoncol.2016.1291 27281650

[34] Neugut AI, Subar M, Wilde ET, . Association between prescription co-payment amount and compliance with adjuvant hormonal therapy in women with early-stage breast cancer. J Clin Oncol. 2011;29(18):2534-2542. doi:10.1200/JCO.2010.33.3179 21606426 PMC3138633

[35] Hershman DL, Accordino MK, Shen S, . Association between nonadherence to cardiovascular risk factor medications after breast cancer diagnosis and incidence of cardiac events. Cancer. 2020;126(7):1541-1549. doi:10.1002/cncr.32690 31913500

[36] Calip GS, Boudreau DM, Loggers ET. Changes in adherence to statins and subsequent lipid profiles during and following breast cancer treatment. Breast Cancer Res Treat. 2013;138(1):225-233. doi:10.1007/s10549-013-2424-2 23358904 PMC3594463

[37] Early Breast Cancer Trialists’ Collaborative Group (EBCTCG). Aromatase inhibitors versus tamoxifen in early breast cancer: patient-level meta-analysis of the randomised trials. Lancet. 2015;386(10001):1341-1352. doi:10.1016/S0140-6736(15)61074-1 26211827

[38] Perez EA. Safety profiles of tamoxifen and the aromatase inhibitors in adjuvant therapy of hormone-responsive early breast cancer. Ann Oncol. 2007;18(suppl 8):viii26-35. doi:10.1093/annonc/mdm263 17890211

