# Supplementary material for: Concomitant Discontinuation of Cardiovascular Therapy and Adjuvant Hormone Therapy Among Patients With Breast Cancer
Source: JAMA Netw Open. 2023 Jul 17;6(7):e2323752. doi: 10.1001/jamanetworkopen.2023.23752 (PMC10352860; doi:10.1001/jamanetworkopen.2023.23752)
Supplement: Supplement 1. — eFigure. Flowchart of Study Population eTable 1. Incidence Rate Ratio and 95% CI of Discontinuing Cardiovascular Therapy Before and After Discontinuation of Adjuvant Hormone Therapy in Breast Cancer Patients, Stratified by Baseline Type of Adjuvant Hormone Therapy eTable 2. Cause-Specific Mortality by Discontinuation of Adjuvant Hormone Therapy in Breast Cancer Patients, Stratified by Baseline Type of Adjuvant Hormone Therapy [file jamanetwopen-e2323752-s001.pdf]

Supplementary Online Content

He W, Zeng E, Sjölander A, Hübbert L, Hedayati E, Czene K. Concomitant discontinuation of cardiovascular therapy and adjuvant hormone therapy among patients with breast cancer. *JAMA Netw Open*. 2023;6(7):e2323752. doi:10.1001/jamanetworkopen.2023.23752

eFigure. Flowchart of Study Population

eTable 1. Incidence Rate Ratio and 95% CI of Discontinuing Cardiovascular Therapy Before and After Discontinuation of Adjuvant Hormone Therapy in Breast Cancer Patients, Stratified by Baseline Type of Adjuvant Hormone Therapy

eTable 2. Cause-Specific Mortality by Discontinuation of Adjuvant Hormone Therapy in Breast Cancer Patients, Stratified by Baseline Type of Adjuvant Hormone Therapy

This supplementary material has been provided by the authors to give readers additional information about their work.

© 2023 He W et al. *JAMA Network Open*.

eFigure. Flowchart of Study Population

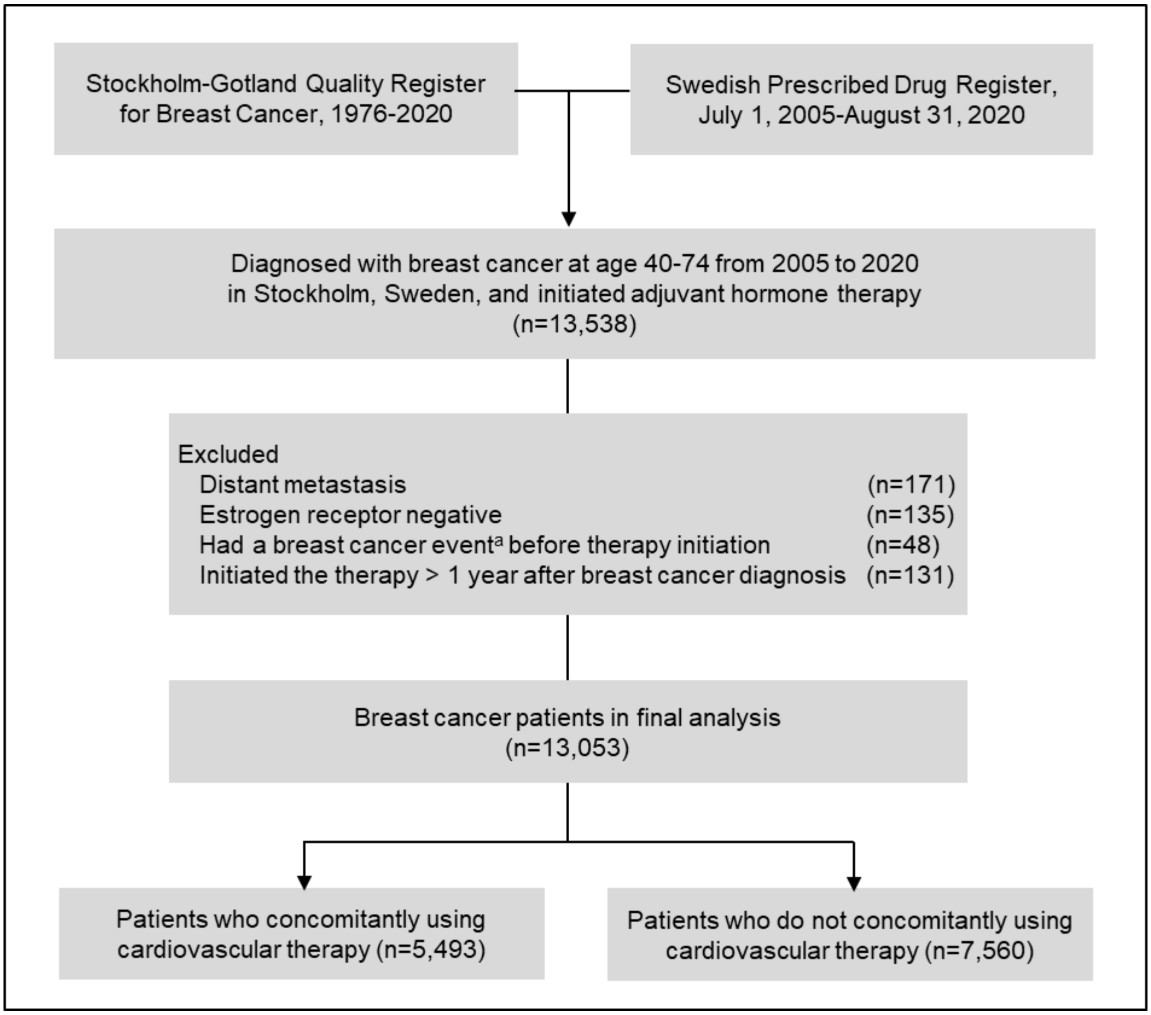

[a] Breast cancer events include local recurrence, distant metastasis, or second primary breast cancer (>3 months after a primary breast cancer diagnosis).

© 2023 He W et al. *JAMA Network Open*.

eTable 1. Incidence Rate Ratio and 95% CI of Discontinuing Cardiovascular Therapy Before and After Discontinuation of Adjuvant Hormone Therapy in Breast Cancer Patients, Stratified by Baseline Type of Adjuvant Hormone Therapy[a]

	Time in relation to adjuvant hormone therapy discontinuation			
	-365 – -91 days	-90 – -1 days	1 – 90 days	91–365 days
**Aromatase inhibitors**				
Discontinuers of adjuvant hormone therapy				
Event	161	113	88	91
Follow-up time (days)	315740	110530	85713	188789
Incidence rate (per 1000 person-day)	0.51	1.02	1.03	0.48
Continuers of adjuvant hormone therapy				
Event	141	57	49	93
Follow-up time (days)	323583	119779	116231	283853
Incidence rate (per 1000 person-day)	0.44	0.48	0.42	0.33
Incidence rate ratio[b]	1.13(0.90-1.43)	2.18(1.55-3.06)	2.42(1.67-3.51)	1.41(1.02-1.96)
**Tamoxifen**				
Discontinuers of adjuvant hormone therapy				
Event	131	60	56	62
Follow-up time (days)	231742	81636	65622	152937
Incidence rate (per 1000 person-day)	0.57	0.73	0.85	0.41
Continuers of adjuvant hormone therapy				
Event	99	47	37	85
Follow-up time (days)	255598	93069	90505	221212
Incidence rate (per 1000 person-day)	0.39	0.51	0.41	0.38
Incidence rate ratio[b]	1.53(1.16-2.00)	1.42(0.94-2.17)	2.20(1.43-3.40)	1.08(0.76-1.53)

[a] Patients who discontinued adjuvant hormone therapy were 1:1 matched to those who continued therapy on breast cancer diagnosis year, age at diagnosis (± 3 years), and type of cardiovascular therapy. Patients were followed from 1 year before adjuvant hormone therapy discontinuation until discontinuation of cardiovascular therapy, 1 year after adjuvant hormone therapy discontinuation, local recurrence, distant metastasis, contralateral breast cancer, cardiovascular comorbidities, death, emigration, completion of 5-year adjuvant hormone therapy or end of the study period (August 31, 2020), whichever came first.

[b] Incidence rate ratio was adjusted for matching variables, tumor size, lymph node status, tumor grade, progesterone receptor status, surgery type, chemotherapy, radiotherapy, baseline hormone therapy type, pre-diagnosis use of cardiovascular therapy, pre-diagnosis major cardiovascular event and Charlson Comorbidity Index at diagnosis.

© 2023 He W et al. *JAMA Network Open*.

eTable 2. Cause-Specific Mortality by Discontinuation of Adjuvant Hormone Therapy in Breast Cancer Patients, Stratified by Baseline Type of Adjuvant Hormone Therapy[a]

	Total	Outcome (10-year mortality)	Adjusted Hazard Ratio (95% CI)
**Breast cancer mortality**			
**Aromatase inhibitors**			
Continuer	1463	65(11.1)	1.00 (reference)
Discontinuer	1509	94(13.8)	1.44(0.97-2.14)
**Tamoxifen**			
Continuer	1152	14(2.2)	1.00 (reference)
Discontinuer	1106	22(3.2)	1.55(0.74-3.21)
**Cardiovascular mortality**			
**Aromatase inhibitors**			
Continuer	1463	29(5.9)	1.00 (reference)
Discontinuer	1509	53(7.7)	1.95 (1. 08-3.54)
**Tamoxifen**			
Continuer	1152	18(3.5)	1.00 (reference)
Discontinuer	1106	27(5.2)	1.64(0.80-3.37)

[a] Patients who discontinued adjuvant hormone therapy were 1:1 matched to those who continued therapy on breast cancer diagnosis year and age at diagnosis (± 3 years). Patients were followed until death, emigration, or end of the study period (December 31, 2019), whichever came first.

[b] Hazard ratio (HR) was adjusted for matching variables, tumor size, lymph node status, tumor grade, progesterone receptor status, surgery type, chemotherapy, radiotherapy, baseline hormone therapy type, pre-diagnosis use of cardiovascular therapy, pre-diagnosis major cardiovascular event and Charlson Comorbidity Index at diagnosis.

© 2023 He W et al. *JAMA Network Open*.
