# Supplementary material for: Concomitant Discontinuation of Cardiovascular Therapy and Adjuvant Hormone Therapy Among Patients With Breast Cancer
Source: JAMA Netw Open. 2023 Jul 17;6(7):e2323752. doi: 10.1001/jamanetworkopen.2023.23752 (PMC10352860; doi:10.1001/jamanetworkopen.2023.23752)
Supplement: Supplement 2. — Data Sharing Statement [file jamanetwopen-e2323752-s002.pdf]

Data Sharing Statement

He. Concomitant Discontinuation of Cardiovascular Therapy and Adjuvant Hormone Therapy Among Patients With Breast Cancer. *JAMA Netw Open*. Published July 17, 2023. doi:10.1001/jamanetworkopen.2023.23752

Data
Data available: No
